# Understanding the cost-utility of implementing HIV self-testing with digital-based supports

**DOI:** 10.3389/fpubh.2024.1440104

**Published:** 2025-01-14

**Authors:** Brianna Empringham, Angela Karellis, Marta Fernandez-Suarez, Sergio Carmona, Nitika Pant Pai, Alice Zwerling

**Affiliations:** ^1^School of Epidemiology and Public Health, University of Ottawa, Ottawa, ON, Canada; ^2^Children’s Hospital of Eastern Ontario, Ottawa, ON, Canada; ^3^Division of Clinical Epidemiology, McGill University, Montreal, QC, Canada; ^4^Foundation for Innovative New Diagnostics (FIND), Geneva, Switzerland

**Keywords:** HIV, Markov model, self-testing, health economics, digital-based intervention

## Abstract

**Introduction:**

HIV self-testing (HIVST) is an innovative strategy that has been shown to increase uptake of HIV testing compared to conventional facility-based testing. HIVST implementation with digital-based supports may help facilitate testing accessibility and linkage to care after a reactive self-test. Economic evidence around community-based implementation of HIVST is growing; however, economic evidence around digital-based HIVST approaches remains limited.

**Methods:**

We used previously published cost and efficacy data from HIVST interventions, with the specific intervention model differing between scenarios. Digital-based interventions included text messaging campaigns and online websites that promoted uptake and linkage to HIVST care. Community-based interventions included door-to-door distribution, peer-incentivized distribution, and mobile testing units. Using data obtained from the literature, we parameterized a combined Markov and decision analytic model to evaluate the cost-utility of digital-based HIVST implementation across Malawi, South Africa, and Brazil compared to both community-based HIVST and facility-based testing.

**Results:**

We found that HIVST was cost-effective compared to facility-based testing in all settings investigated. Our scenarios predicted that digital-based HIVST was associated with an incremental cost in the range of $769–$17,839/DALY (disability-adjusted life year) averted compared to facility-based testing across Malawi, South Africa, and Brazil. Digital-based HIVST cost savings had an incremental cost of $7,300/DALY averted compared to community-based HIVST. The main drivers of cost-utility included HIV test and treatment costs, HIV test-positivity, rates of linkage to care, and antiretroviral therapy (ART) initiation rates. Digital-based supports were associated with an increased cost compared to facility-based testing, but they also had increased utility, which led to favorable cost-utility estimates.

**Discussion:**

HIVST with digital supports has the potential to be a highly cost-effective approach, with the potential to make HIV testing more available and accessible, thereby increasing overall uptake and coverage of HIV testing. Digital supports can also support linkage to care, which we have identified as a major driver of cost-utility. Strategies to improve cost-utility include reducing testing costs, targeting key populations with increased rates of HIV test-positivity, and ensuring strong support for linkage to care.

## Introduction

The United Nations General Assembly has established ambitious goals for HIV diagnosis and care to be met by the end of 2030 (1). Although global progress has been made, many countries have yet to attain critical targets (2–4). There have been significant advances in diagnostic technologies and in novel treatment and supportive care options for HIV (5).

An innovative approach to promote HIV screening and diagnosis is HIV self-testing (HIVST), where individuals can perform their own HIV screening. HIVST provides a private and anonymous method for testing that is perceived by users as more convenient than accessing testing through conventional health clinics (6, 7). Self-testing is a highly promising strategy with the potential to reduce the global HIV burden by bringing the currently undiagnosed to care (8). Previous literature strongly suggests that HIVST is preferred by clients with higher rates of uptake compared to conventional testing. However, there are many factors that affect testing outcomes (9–11). A recent systematic review found that HIVST had a variable uptake rate of 20–92% depending on the implementation strategy and specific subpopulation being tested (6).

There are many advantages to HIVST; however, concern has been concern raised regarding the linkage to care, with some studies suggesting that linkage to care may be lower with HIVST (particularly with home-based approaches) (12–14). Varoious strategies have been used to implement HIVST and support and improve uptake and linkage to care, including pairing HIVST with community-based and digital-based supports. HIVST with community-based support involves utilizing resources from within the community, such as existing health infrastructure, volunteers, or peer support groups, to support uptake and linkage to care (15). HIVST with digital-based support typically includes the use of digital interventions such as SMS messaging, websites, downloadable applications, or social media to improve uptake, help with user experience and ease of testing, provide counseling and help assist with clinical care and follow-up. As access to the internet improves globally (16), HIVST with digital support offers improved accessibility and confidentiality for HIVST, especially among hard-to-reach populations (17) or stigmatized groups (7).

Population-level screening programs can be resource-intense, and economic evidence is critical in providing evidence to guide implementation and scaling up of such programs. Previous studies have strongly supported that HIVST is cost-effective among a broad range of contexts, primarily using community-based distribution approaches. Matsimela et al. (18) conducted a micro-costing and economic evaluation of eleven community-based HIVST distribution strategies in South Africa. They observed that HIVST distribution strategies across South Africa varied significantly by volume distributed, cost per kit, underlying test positivity of HIV in the population tested, and rates of linkage to care. These factors contributed to the wide range of cost-effectiveness estimates between distribution strategies ($5–19 USD/person tested, $61–1,277 USD/person diagnosed).

HIVST has two published interventions using digital-based supports that include both costing and outcome measures. Both studies reported cost-effectiveness; however, cost-utility measures were not evaluated. Kelvin et al. used SMS-based messaging to target truck drivers and female sex workers for their HIVST campaign (19, 20). They found that HIVST was preferable compared to facility-based testing (FBT) and that it improved overall rates of HIV testing. HIVST was associated with a cost of $10.13/person tested, compared to $5.01 for FBT (20). Because they did not report diagnostic outcomes, costs per diagnosis were unavailable. DeBoni et al. (21) evaluated an HIVST intervention using website-based supports in Brazil. They found that digital-based HIVST was associated with a cost of $176/person tested or $15,717/person diagnosed (21, 22).

To our knowledge, this is the first model evaluating the cost-utility of a digital-based implementation of HIVST. Cost-utility implies that costs are reported per utility measure, which includes both duration and quality of life impacted, compared to cost-effectiveness, which reports cost per outcome (e.g., cost per quality-adjusted life year versus cost per person diagnosed). The aim of this study is to evaluate the cost-utility of HIVST using digital-based (DB) supports compared to HIVST using community-based (CB) supports or facility-based testing (FBT) alone.

## Methodology

### Design and setting

We evaluated the cost-utility of implementing HIVST using digital-based modalities compared to two comparator arms, namely HIVST using a community-based approach or FBT alone. We considered FBT to be the current standard of care and therefore modeled HIVST so that it was implemented in addition to the standard of care rather than replacing FBT.

**Figure 1:**
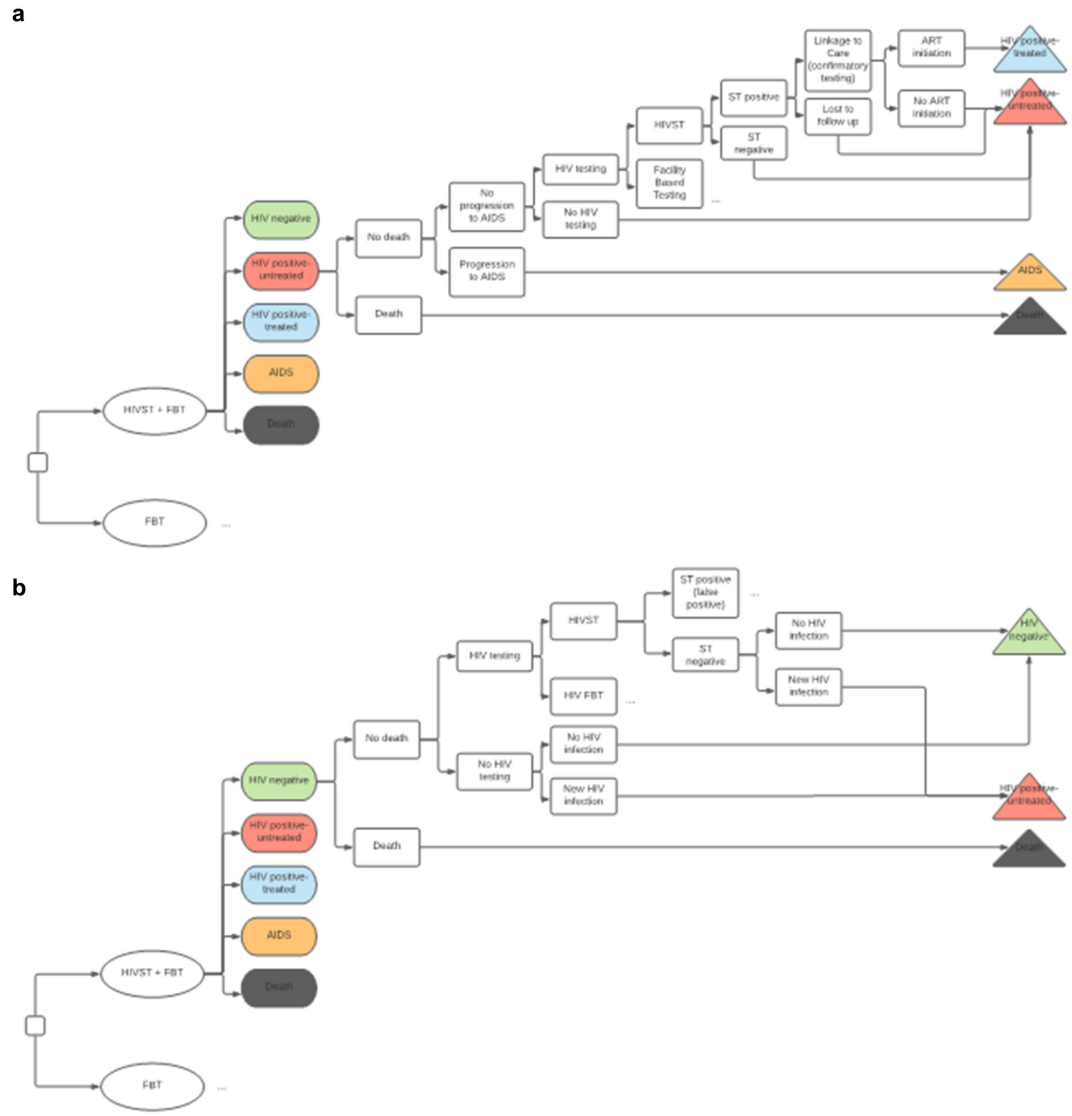
**(A)** Decision Tree for HIV positive Markov state. HIVST, HIV self-testing; FBT, facility-based testing; AIDS, acquired immunodeficiency syndrome; ST, self-testing; ART, antiretroviral therapy. Description: This diagram depicts the combined decision tree and Markov structure of the economic model. We have compared HIVST and FBT (the standard of care) in addition to FBT alone. The color-coded ovals in this diagram represent Markov states. A cohort progresses through the decision tree structure with every Markov cycle, and a proportion of the cohort may progress to a different Markov state, as illustrated by the terminal triangles. This diagram shows how PLHIV who are untreated may get transitioned to starting ART. **(B)** Decision tree for HIV negative Markov state. HIVST, HIV self-testing; FBT, facility-based testing; AIDS, acquired immunodeficiency syndrome; ST, self-testing; ART, antiretroviral therapy. Description: this diagram illustrates how individuals can transform from the HIV negative stage to the HIV positive stage. Depending on the specific test-positivity, a percentage of the population entered the model in the HIV positive-untreated state. This reflects the HIV test positive rate of the cohort. The other individuals entered the model with HIV negative state. With every cycle, there is a small probability of new HIV infection, where individuals who are HIV negative can move to the HIV positive-untreated state.

**Figure 2:**
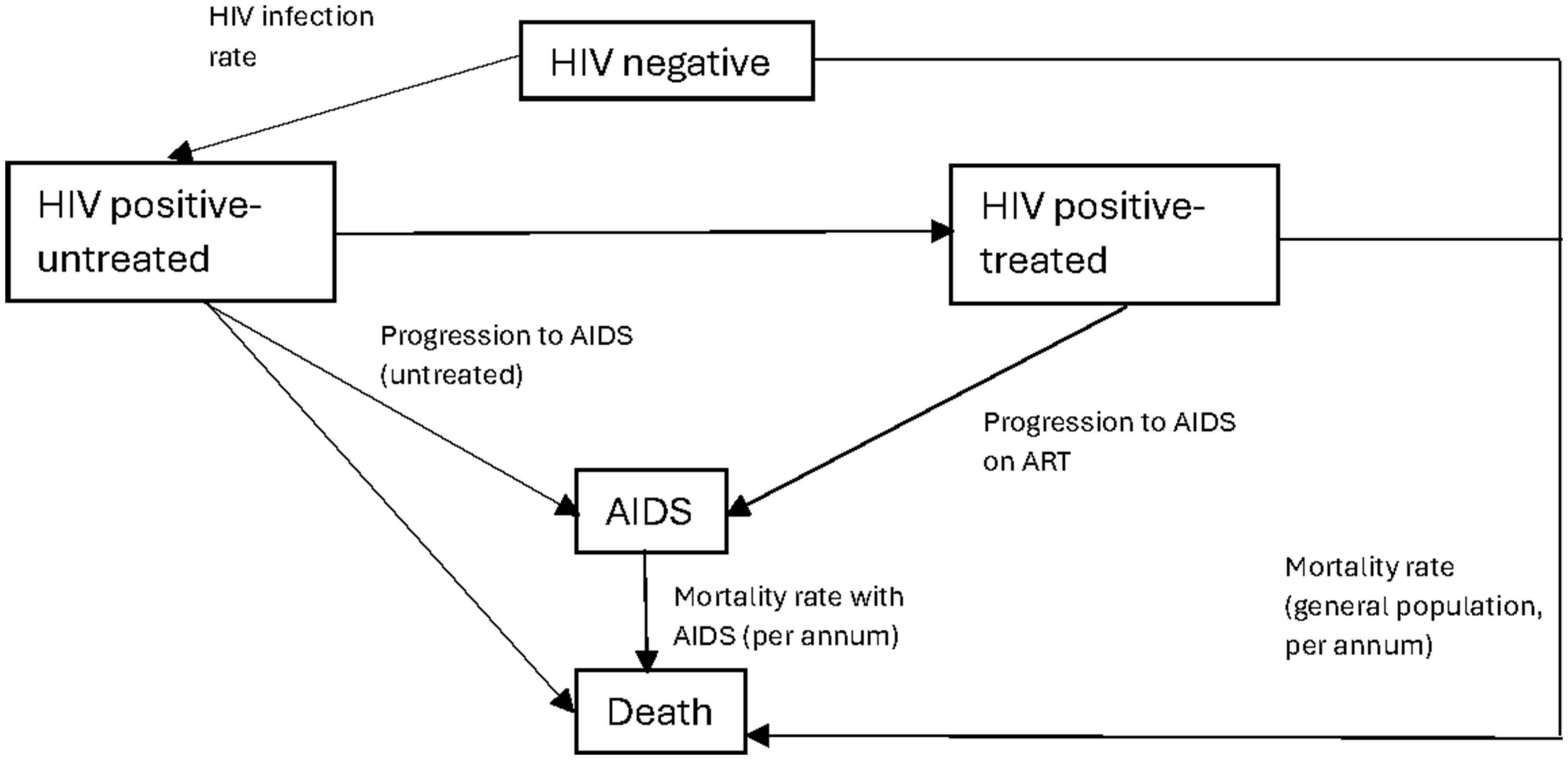
Markov model schematic. AIDS, acquired immunodeficiency syndrome; HIV, human immunodeficiency virus; ART, antiretroviral therapy. Description: this figure shows the general schema of the Markov model and how the cohort moves between different Markov states. The transition probabilities between states are depicted on the diagram. The corresponding quantitative values are included in Table 2. Individuals who enter the model are either the HIV positive (undiagnosed) or HIV negative. With each Markov cycle, there exists a transitional probability to undergo HIV testing. For individuals who are diagnosed, they can either be treated or choose not to start ART. With each cycle, there is a transitional probability of a new HIV infection or death. The probability of death is dependent on the Markov state, with those within the AIDS Markov state having a higher probability of death.

We developed an embedded decision tree within a Markov model structure using TreeAge Pro 2021 (23). We used a decision tree, with mutually exclusive branches, to capture the diagnostic testing process, confirmatory testing, and linkage to care (Figures 1A,B) and a Markov model for the chronic health states of HIV positive (on and off antiretroviral therapy), HIV negative, and death (Figure 2). The Markov cycle duration was one year and the time horizon was 30 years, which was varied from 5 to 50 years in sensitivity analysis. This implies that the intervention modeled incurred costs and benefits for a period of thirty years. The time horizon will impact cost (which accumulates yearly), but it will also impact the ability to detect benefits of screening programs on disease-related morbidity and mortality, especially in infections like HIV, which have a chronic course. We modeled the cost-utility of HIVST across three countries—Malawi, South Africa, and Brazil. These countries were selected to represent a variety of income levels and endemic HIV rates.

We reported our findings based on the CHEERs (Consolidated Health Economic Evaluation Reporting Standards) checklist for model-based economic evaluations (24).

### Patient population

We modeled the cost-utility of HIVST assuming a general population (ages 15–65 years) who had not previously tested positive for HIV. We also included scenario analyses where HIVST was restricted to key populations, such as men who have sex with men (MSM), with higher underlying rates of test positivity for HIV. PrEP was included in our model, with the percentage of the population on PrEP based on existing literature (25–27). Individuals on PrEP were eligible for HIVST and FBT annually and were subject to incurred regular testing and treatment costs as per country-specific guidelines. Individuals on PrEP incurred additional treatment costs but had a greatly decreased risk of becoming HIV positive (28–30).

### Interventions of interest and comparator

Our primary intervention of interest was HIVST using a digital-based approach, which was implemented in addition to the current standard of care, which consisted of HIV testing offered at a clinic (FBT). We used pre-existing literature evaluating digital-based HIVST, community-based HIVST, and facility-based HIV testing to inform cost and effectiveness parameters within the model (19–22, 31, 32). Those with a reactive self-test required follow-up (linkage to care) with confirmatory testing and post-test counseling provided at a health care facility. The costs associated with confirmatory FBT from true and false positive HIVST screens were attributed to the intervention arm. Linkage to care rates were varied depending on the HIVST implementation strategy. FBT required an individual to present to the health care facility for screening and confirmatory testing as per the current standard of care based on country-specific HIV confirmatory testing guidelines (33–35). After confirmatory testing (in both the HIVST and FBT scenarios), individuals could be initiated on antiretroviral therapy (ART). Rates of ART acceptance rates were country-specific, based on published literature (6, 7, 36–38).

### Model parameters

Key epidemiological parameters such as HIV test-positivity, HIV incidence, and population mortality were sourced from the national databases (Table 1). Our model accounted for the rate of new HIV infections (HIV incidence) by using country-specific infection rates (39). Individuals who were HIV negative experienced an annual rate of transition to become HIV positive based on the HIV infection rate. The underlying HIV test-positivity rates were sourced from the UNAIDS (Joint United Nations Programme on HIV and AIDS) data bank (4) and were country and age specific.

**Table 1:** Country specific epidemiological inputs.

	HIV test positive probability (proportion)	HIV infection probability (*per annum*) (39)	Mortality probability (*per annum*) (70)	PrEP proportion (%)	WTP threshold for high cost-effectiveness: GDP *per capita* (56) (USD per DALY averted)	WTP threshold for cost-effectiveness: 3x GDP *per capita* (56) (USD per DALY averted)
Malawi	0.09 (15, 71, 72) (0.084–0.096) DA: 0.01–0.15	0.0029 (0.0027–0.0031) DA: 0.001–0.004	0.006 (0.0056–0.0064) DA: 0.001–0.01	1.10 (25–27) (0.8–1.5) DA: 0–4	410	1,230
Brazil	0.003 (73, 74) (0.0029–0.0030) DA: 0.001–0.01	0.0022 (0.0021–0.0023) DA: 0.001–0.003	0.0065 (0.0059–0.0073) DA: 0.005–0.01	1.10 (25–27) (0.8–1.5) DA: 0–4	8,717	26,152
South Africa	0.08 (75, 76) (0.068–0.093) DA: 0.01–0.15	0.0034 (0.0032–0.0036) DA: 0.0015–0.005	0.009 (0.0086–0.0094) DA: 0.005–0.01	1.10 (25–27) DA: 0–4	6,001	18,003

HIV; human immunodeficiency virus; PrEP, pre-exposure prophylaxis; WTP, willingness to pay; GDP, gross domestic product; USD, US dollars; DALY, disability-adjusted life year; DA, deterministic analysis.

Ranges represent 95% confidence intervals, varied in probabilistic analysis.

Deterministic analysis represents the range of feasible values that were varied in threshold analysis to determine the impact on cost-utility estimates (tornado diagrams are included in appendices).

The specific HIV self-test model was saliva-based OraQuick™, given that it is the HIV self-test that has been most commonly implemented on a global basis and for which there is existing economic literature in the context of HIVST (40–44).

To model digital and community-based supports, we used data from previously published interventional studies to parametrize each country-specific model (Table 2). In order to identify all relevant literature, a comprehensive literature review focusing on economic studies for HIVST (both community and digital-based) was conducted (45). For our Malawi digital-based (DB) model, parameters relevant to the DB intervention, including cost, uptake, and linkage to care, were sourced from a randomized control trial conducted in Kenya, using SMS-based messaging to target truck drivers and female sex workers (FSWs) for HIVST (19, 20). In Malawi, there are no current publications on the cost-effectiveness of DB HIVST, which is why a Kenyan study was chosen.

**Table 2:** Self-testing parameters for country-specific scenarios.

Scenario	South Africa-CB	South Africa-DB	Malawi- CB	Malawi-DB	Brazil-CB	Brazil-DB
HIVST cost (USD 2020/ST)	37.7 (60) (35.20–40.15) DA: 20–250	99.58 (21, 22) (92.10–107) DA: 50–250	5.7 (15) (5.2–6.2) DA: 2–50	12.96 (20) (12–13.9) DA: 2–50	52.1 (22) (43.2–60.1) DA: 20–250	128 (21, 22) (116.4–140.2) DA: 50–500
FBT cost (USD 2020/FBT)	5.68 (45) (2.75–15.28) DA: 2–50	5.68 (45) (2.75–15.28) DA: 2–50	5.68 (45) (2.75–15.28) DA: 2–50	5.68 (45) (2.75–15.28) DA: 2–50	5.68 (45) (2.75–15.28) DA: 2–50	5.68 (45) (2.75–15.28) DA: 2–50
CB/DB intervention	HIVST is available through mobile testing units (60, 77)	Website promoting HIVST with online ordering (21)	Volunteer delivery of HIVST to homes within the community (15)	Text messages to the general population promoting HIVST (19, 31, 32)	HIVST is available through mobile testing units (22)	Website promoting HIVST with online ordering (21)
Proportion of population undergoing FBT	0.15 (45)	0.15 (45)	0.15 (45)	0.15 (45)	0.15 (45)	0.15 (45)
HIVST uptake (%)	92 (77) (84–97) DA: 5–100	21.4 (21) (19–23) DA: 5–100	100 (15) (88–100) DA: 5–100	32.7 (19, 31, 32) (29.6–35.8) DA: 5–100	49 (22) (45.2–53.4) DA: 5–100	21.4 (21) (18.7–23.4) DA: 5–100
Linkage to care (%)	58 (77) (56–61) DA: 20–100	80 (21) (72.3–88.2) DA: 20–100	75 (15) (71.9–78.1) DA: 20–100	85 (75.7–94.3) (19, 31, 32) DA: 20–100	23 (22) (20.1–26.5) DA: 20–100	80 (21) (76.3–84.3) DA: 20–100
ART initiation (%)	78.4 (7, 8, 37, 69) (71–85.8) DA: 50–100	78.4 (7, 8, 37, 69) (71–85.8) DA: 50–100	70 (36, 78) (62.6–77.4) DA: 50–100	70 (36, 78) (62.6–77.4) DA: 50–100	80 (74, 79, 80) (73.1–87.3) DA: 50–100	80 (74, 79, 80) (73.1–87.3) DA: 50–100 DA: 50–100

CB, community-based; DB, digital-based; HIVST, HIV self-testing; ART, antiretroviral therapy; USD, US dollars; ST, self-test.

This table shows the inputs extracted from previously published literature that parameterized the model for our country-specific scenarios. For the Malawi community-based intervention, HIVST were delivered to all the homes within the community; therefore, the uptake was estimated at 100%.

We parameterized our model with effectiveness data from a published study that used a digital application to promote HIVST in South Africa along with counseling and linkage services (46). We extrapolated costing data from Brazil to estimate the cost-utility of DB HIVST in South Africa, as there is no published cost-effectiveness data for DB HIVST in South Africa. For both Malawi and South Africa, where there were no country-specific costing data available for DB HIVST, a corrective ratio was applied based on GDP *per capita*.

The DB intervention from Brazil is based on a study conducted by DeBoni et al. (21), using a website targeted at the MSM population to facilitate HIVST. For further details of the CB or DB HIVST intervention used in each scenario, we have included detailed summaries of the studies used to parameterize our model in our appendices.

### Economic approach

The cost per HIV self-test (including unit test and implementation cost) was sourced from the relevant published literature for both digital and community-based HIVST (Table 2). For the comparator arm (facility-based testing), the average cost per HIVST ($5.68/test) was used based on a literature heterogeneity of all available published economic data for HIVST. Unweighted mean costs were employed and sourced from the 22 relevant studies identified by the literature review (45). We assumed individuals who were HIV positive and had initiated ART would have incurred annual ART and monitoring costs that were sourced from country-specific literature (47–49).

We have adopted a health care system perspective since there was inadequate data to inform patient incurred costs around HIVST. A mid-cycle correction was applied to the costs and utilities. Both costs and utilities are discounted by 3% *per annum*. All costs were converted back to their original currency (50), adjusted for inflation (51, 52) and then converted into USD 2020.

### Outcomes

The primary outcome evaluated was the incremental cost-effectiveness ratio (ICER), defined as the incremental cost in USD per disability-adjusted life year (DALY) averted (53). Values for disability weights were sourced from the WHO Global Burden of Disease study (54). ICER calculations were conducted with digital-based testing compared to both community-based HIVST and FBT only. ICER per DALY averted was used as a primary outcome because it is a standardized measure that allows comparison between studies and accounts for quality and quantity of life.

The ICER estimates were compared against a willingness to pay threshold (WTP) established *a priori* to determine whether the intervention should be considered cost-effective. As per the WHO Choosing Interventions that are Cost-Effective (CHOICE) guidelines (55), the intervention could be considered cost-effective if it is less than three times the national gross domestic product (GDP) *per capita* per DALY averted and highly cost-effective if it is less than the national GDP *per capita* (56).

### Sensitivity analyses

Uncertainty around parameter values was explored through one- and two-way deterministic sensitivity analyses to understand the impact of key parameters varied along plausible ranges on model results and to identify key drivers of cost-effectiveness. Key parameters identified comprised of test positivity, cost of HIVST, HIVST uptake, linkage to care, and ART initiation rates (57–60). We varied the horizon from 5 to 50 years to understand the impact of intervention duration on cost-utility estimates.

A probabilistic sensitivity analysis was conducted using 10,000 Monte Carlo repetitions to generate 95% uncertainty ranges around model point estimates. Costs are represented by gamma distributions, transitional probabilities are modeled by beta distributions, and utility values are represented by triangular distributions.

As the HIVST unit costs employed in the model included costs associated with program implementation costs as well as individual kit costs, we sought to explore the further benefits associated with economies of scale wherein implementation costs could be spread over different target population sizes. To address this, a scenario analysis was performed where the programmatic portion of HIVST is divided by an increasing number of people to evaluate how increased uptake leading to reduced unit test costs might affect model estimates. The scale-up factor for this sensitivity analysis was varied from 0.5 to 10x.

## Malawi

### Results

**Figure 3:**
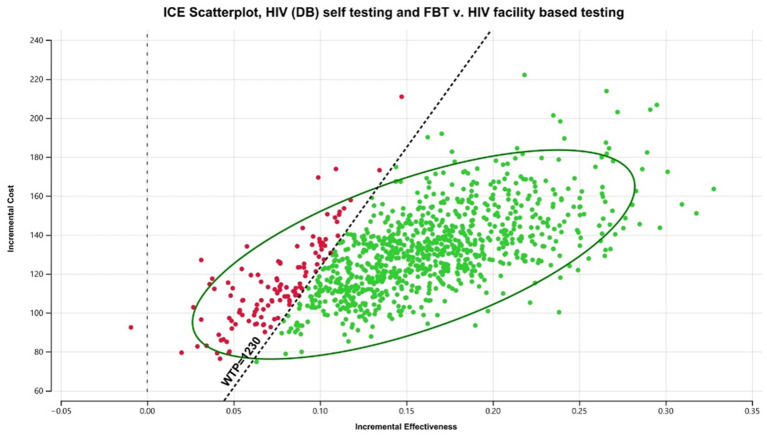
Probabilistic analysis for Malawi digital-based HIVST compared to FBT. HIVST, HIV self-testing; GDP, gross domestic product; WTP, willingness to pay; USD, US Dollars; DALY, disability-adjusted life year; CB, community-based. Description: WTP = 3x Malawian GDP *per capita*.

In the Malawian scenario, digital-based HIVST was more cost-effective compared to FBT, but it was associated with a lower ICER than CB HIVST. DB HIVST was associated with an ICER of $769/DALY averted compared to facility-based testing. We have discovered that DB HIVST was less effective but cost-saving compared to community-based testing. Community-based testing was associated with an additional cost of $400/DALY averted compared to digital-based HIVST. Using a WTP threshold of 3x Malawian GDP *per capita*, the digital-based HIVST was cost-effective in 88% of the probabilistic scenarios (Figure 3). HIVST with DB supports was associated with a total cost of $10.13/person tested *per annum*, which includes testing costs, programmatic costs, and treatment for the additional people diagnosed with HIV. If digital-based HIVST was implemented on a national level in Malawi (among a population of approximately 19.9 million), it would be associated with an additional 318,400 individuals initiating ART and 278,600 deaths averted compared to FBT. Compared to FBT, digital-based HIVST with FBT was associated with a 32% increase in ART initiation among people living with HIV (PLHIV).

The drivers of cost-utility for digital-based HIVST in Malawi were the cost of the HIVST, HIV test-positivity rates, linkage to care, and annual ART costs (Appendix Figure 1). In threshold analysis, the minimum linkage to care required for DB HIVST to be considered cost-effective was 35%.

In order to examine the impact of targeting the intervention to key subgroups with increased rates of HIV test positivity, we modeled the cost-utility of a DB intervention limited to the MSM population (4, 61, 62). We found that DB HIVST was more cost-effective within this subgroup, with a decreased ICER of $511/DALY averted. This would be considered cost-effective (costing less than 3x the Malawian GDP *per capita*).

#### South Africa

**Figure 4:**
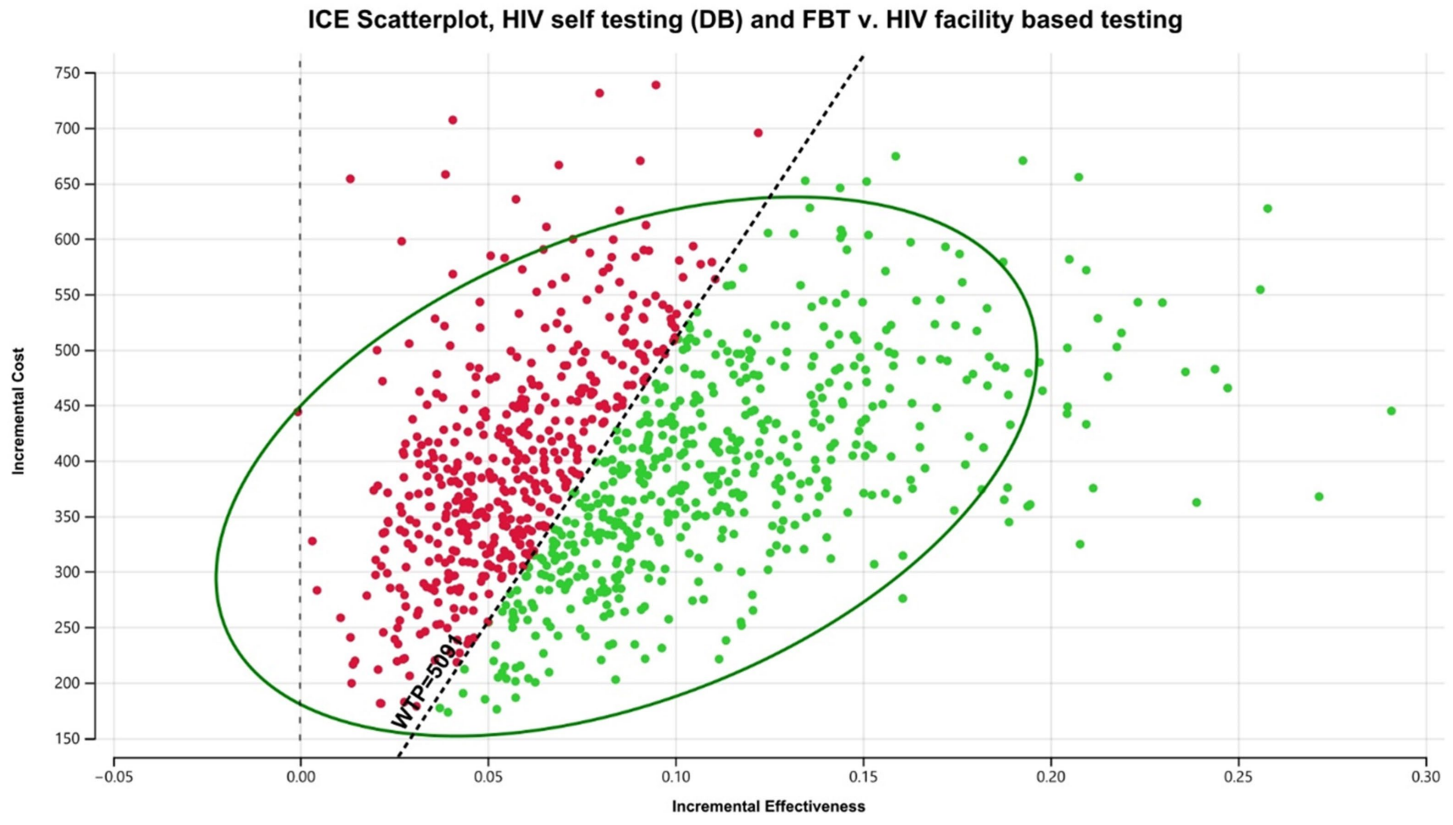
Probabilistic analysis for South Africa digital-based HIVST compared to FBT. HIVST, HIV self-testing; GDP, gross domestic product; WTP, willingness to pay; USD, US dollars; CB, community-based. Description: WTP = 1 x South African GDP *per capita*.

In the South African scenario, DB HIVST was considered highly cost-effective compared to FBT, with an ICER of $4,584/DALY averted (Table 3). However, it was associated with a lower ICER than CB HIVST. Probabilistic analysis showed that the chance that HIVST would be highly cost-effective when implemented through a digital-based approach was 55% (Figure 4). If we used the less conservative WTP threshold of 3x GDP *per capita*, DB HIVST would have >99% chance of being cost-effective compared to FBT. Our model predicts that DB HIVST would be less effective but more economical compared to CB HIVST. CB HIVST was associated with an additional cost of $3,933/DALY averted compared to DB HIVST. HIVST with DB supports was associated with a total cost of $152.81/person tested *per annum*. If DB HIVST was enacted at a national level in South Africa (among a population of approximately 59.4 million), it would be associated with an additional 356,340 individuals starting on ART and 297,000 deaths averted. Compared to FBT, digital-based HIVST with FBT was associated with a 20% increase in ART initiation among PLHIV.

**Table 3:** Cost-effectiveness results by country.

	Cost (USD, 2020)	Incremental Cost (USD, 2020)	Effectiveness (DALYs averted)	Incremental effectiveness (DALYs averted)	ICER (incremental cost/incremental effectiveness)
Malawi					
FBT	168	-	17.27	-	
DB HIVST + FBT	304	135	17.45	0.18	768.66
CB HIVST + FBT	336	168	17.53	0.26	651.40
South Africa					
HIV FBT	153	-	16.98	-	-
DB HIVST + FBT	546	394	17.07	0.09	4584.35
CB HIVST + FBT	782	630	17.13	0.15	4245.39
Brazil					
HIV FBT	454	-	18.19	-	-
CB HIVST + FBT	998	544	18.22	0.02	23874.58
DB HIVST + FBT	1,071	617	18.23	0.03	17839.25

FBT, facility-based testing; HIVST, HIV self-testing; USD, US dollars; DALYs, disability adjusted life years; ICER, incremental cost-effectiveness ratio; MSM, men who have sex with men; DB, digital-based; CB, community-based.

Interventions are listed in order of increasing effectiveness.

In sensitivity analyses, the major driver of cost-utility was the rate of HIV test positivity (Appendix Figure 2). In threshold analysis, DB HIVST continued to be cost-effective throughout a widely varied linkage to care rate (20–100%).

When DB HIVST was targeted toward MSM, we found that HIVST with a DB support was more cost-effective with a decreased ICER of $2,871/DALY averted. This would be considered highly cost-effective (costing less than the South African GDP *per capita*).

#### Brazil

**Figure 5:**
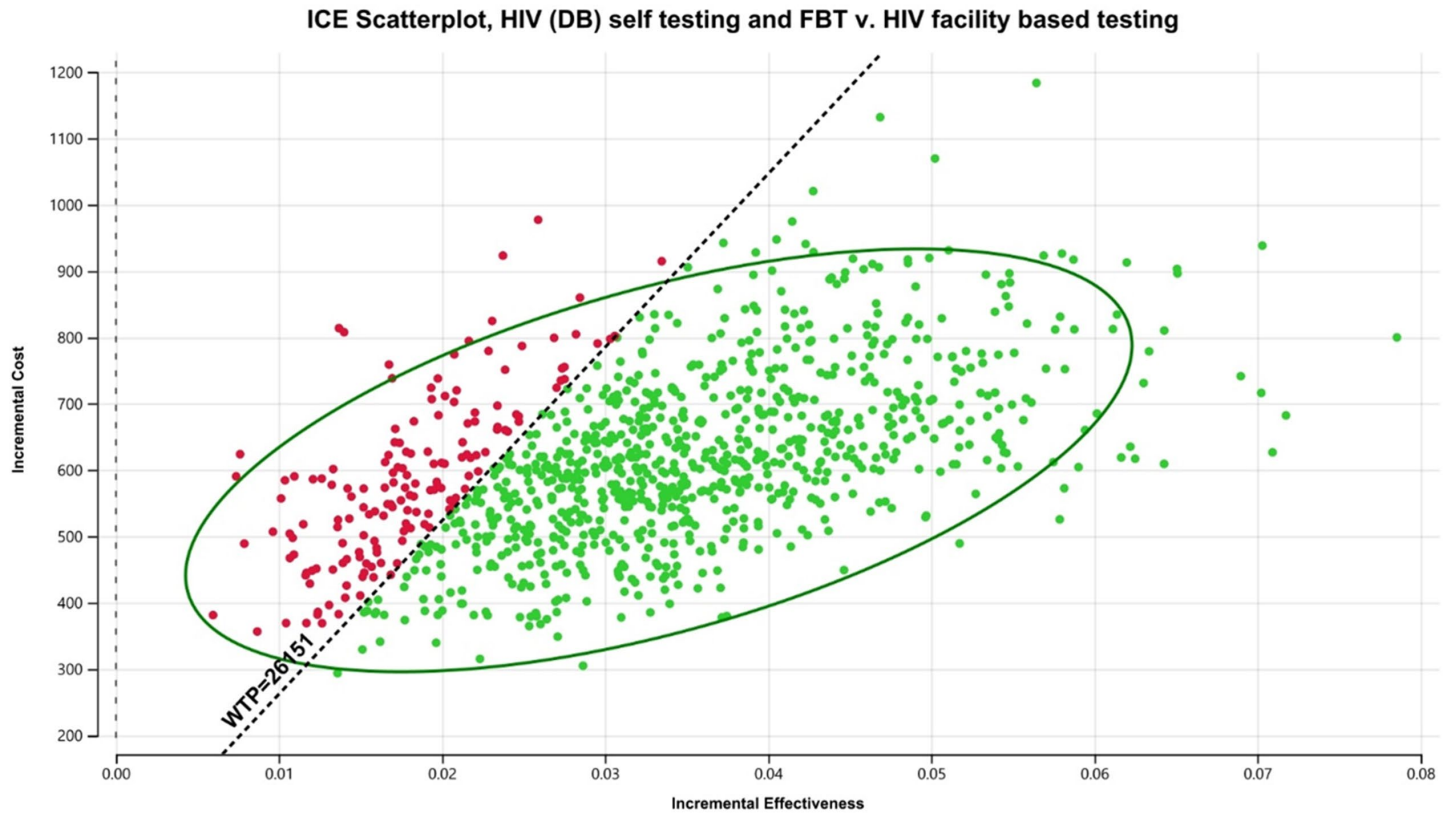
Probabilistic analysis for Brazil digital-based HIVST compared to FBT. HIVST, HIV self-testing; GDP, gross domestic product; WTP, willingness to pay; USD, US dollars; DB, digital-based. Description: WTP = 3 x Brazilian GDP *per capita*.

Within Brazil, DB HIVST would be considered cost-effective with an average ICER of $17,839/DALY averted compared to FBT. Digital-based HIVST was more cost-effective than CB HIVST. A probability analysis indicated that DB HIVST would have an 82% probability of being cost-effective (Figure 5). Within this context, DB HIVST was more expensive and more effective than CB HIVST with an ICER of $7,300/DALY averted. HIVST with DB supports was associated with a total cost of $594.64/person tested *per annum*.

If digital-based HIVST was enacted on a national level in Brazil (among a population of approximately 214.3 million), it would be associated with an additional 1.5 million individuals starting on ART and 857,200 deaths averted. When compared to FBT, digital-based HIVST with FBT was associated with a 33% increase in ART initiation among PLHIV.

The key determinants of cost-utility within this scenario were the cost of HIVST and linkage to care (Appendix Figure 3). In threshold analysis, the minimum linkage to care for DB HIVST to be considered cost-effective was 48%.

When DB HIVST was targeted toward MSM, we noticed that HIVST with a DB support was more cost-effective, resulting in a decreased ICER of $5,703/DALY averted. It would be considered highly cost-effective (costing less than the Brazilian GDP *per capita*). Increasing uptake of PrEP among this population reduce the cost-effectiveness of HIVST by decreasing HIV infection rates and underlying seropositivity of the group.

### Scenario analysis: horizontal duration

We found that the cost-utility of HIVST improved with an increased horizon (Table 4). The cost-utility of HIVST improved with decreased horizon despite decreased intervention costs because the long-term benefits in DALYs averted were properly captured. HIVST continued to be increasingly cost-effective up to a 50-year horizon; however, the rate of improved cost-utility plateaued between 30 and 50 years.

**Table 4:** Impact of varying horizons on cost-utility.

Horizon	Brazil (ICER, USD per DALY averted)	South Africa (ICER, USD per DALY averted)	Malawi (ICER, USD per DALY averted)
5 years	226,177	11,970	2,886
10 years	70,531	6,587	1,436
30 years	17,839	4,584	769
50 years	11,963	3,969	556

ICER, incremental cost-effectiveness ratio; DALY, disability-adjusted life year; USD, US dollars.

### Scenario analysis: HIVST uptake

**Figure 6:**
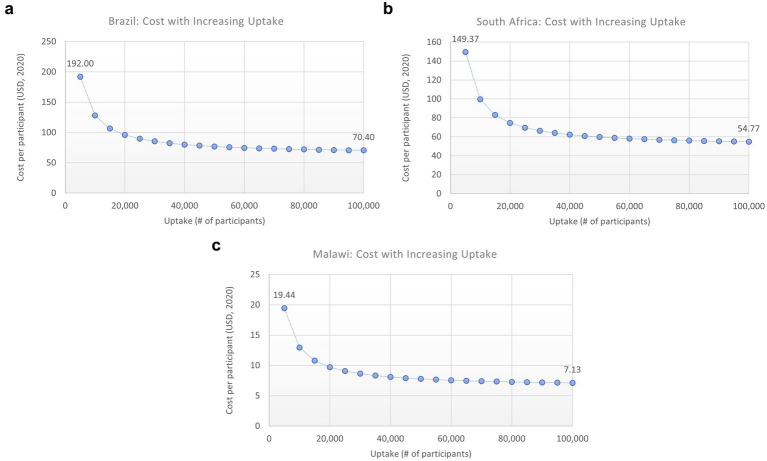
**(A–C)** Impact of increased uptake on cost/person. HIVST, HIV self-testing; ICER, incremental cost-effectiveness ratio; USD, US Dollars. Description: this graph shows how the ICER would change for each scenario with increasing uptake (number of participants), which would decrease the overhead cost per person tested.

To evaluate the potential impact of economies of scale on unit cost per person tested and cost-effectiveness, we performed a sensitivity analysis in which a proportion of the HIVST cost, representing the implementation or “one-time programmatic” costs, was divided among an increasing number of participants. This analysis showed that as uptake increased, the cost per person tested decreased, and HIVST became increasingly cost-effective (Figures 6A–C).

## Discussion

Our model suggests that digital-based HIVST in addition to FBT resulted in more new diagnoses than FBT alone, but that it was associated with an additional cost that varied by implementation approach. In all the scenarios evaluated, digital-based HIVST was considered cost-effective compared to FBT. Compared to community-based HIVST, digital-based HIVST ranged from cost-saving to $7,300/DALY averted. The cost-utility of digital-based HIVST varied between scenarios, with an average ICER/DALY averted ranging from $769 to $17,839 compared to facility-based testing. The upper estimate for this range comes from HIVST in Brazil, where testing and treatment costs were significantly higher compared to Malawi and South Africa.

Compared to facility-based testing, CB HIVST was more cost-effective in Malawi and South Africa than DB HIVST but less cost-effective in Brazil. In Brazil, costs for DB supports were much lower than CB supports, which drove the superior cost-utility profile of DB HIVST in the Brazilian setting. In addition, linkage to care for the CB HIVST was lower than DB HIVST (23% vs. 80%) within the Brazilian setting, which was also a major determinant of cost-effectiveness. Rates of linkage to care are highly variable within the published literature for HIVST. When we compared linkage to care rates from 20 to 100% through deterministic analysis, the DB HIVST and CB HIVST cost-utility estimates overlapped (Appendix Figures 1–3).

The major drivers of cost-utility in our model were largely consistent across different scenarios investigated and highlighted the importance of linkage to confirmatory testing and care, underlying HIV test-positivity, acceptability of ART, and cost of the HIVST test. By modeling previously published DB HIVST interventions on a national scale, using country-specific epidemiological inputs, we were able to enhance the conclusions from prior studies and increase the generalizability of results.

A recent systematic review suggested that HIVST, especially when implemented without support for follow-up, may be associated with lower linkage to care compared to FBT (63). By modeling different rates of linkage to care, we were able to determine the proportion of individuals who would need to link to care to meet cost-utility thresholds in different settings. In the scenarios evaluated, the minimum linkage to care to meet cost-utility thresholds was 20–48%. This is a critical element for consideration by programs when implementing or scaling up self-test approaches.

Although our model suggests that digital-based supports for HIVST are likely to be cost-effective, they require the user to possess device and/or internet access. In January 2023, it was estimated that 64% of the global population had access to the internet, and this has been increasing rapidly (56). Despite improvements, the lack of uniform devices and internet access could lead to gaps in coverage and impact uptake of DB HIVST. We anticipate that HIVST using digital-based supports serves as an adjunct rather than a replacement for facility-based diagnosis. This is why the interventional arm of our Markov model included both digital-based and facility-based testing, compared to only facility-based testing alone. If resources were re-allocated to the DB HIVST from the current standard of care (FBT), then it would pose a health equity concern for individuals without access to devices or the internet.

This is the initial model to estimate cost-utility outcomes [in either DALYs averted or quality-adjusted life years (QALYs)] for digital-based programs. For our CB HIVST results, our estimates were similar to previously published literature once results were adjusted for inflation. Cambiano et al. estimated that CB HIVST was associated with a cost-utility of $23-418/DALY averted when implemented among high-risk populations in Malawi (58). Our estimate for the general population (with a lower test-positivity rate) was $651/DALY averted compared to FBT. Maheswaran et al. also modeled the cost-utility of CB HIVST but they used QALYs as their outcome measures (64). The lack of generalizability across studies suggests a need for reporting standardization among economic modeling studies.

To allow for generalization, we have combined a variety of interventions into the category of digital-based HIVST. It is important to recognize that different programs may employ different digital strategies employed and have a different cost-utility profile. There was limited costing data around digital-based HIVST, with only two relevant studies providing cost data for DB interventions. All current studies have focused on key populations with high underlying rates of HIV test positivity. This would be an important area for further research moving forward, given the increasing digitalization of health care on a global level. Our findings highlight the emerging opportunity to use digital-based support in conjunction with HIVST to help testing become more accessible to communities and support linkage to care, especially among those who face barriers to accessing health care through conventional means. This could serve as an effective means to improving the accessibility of HIVST among currently “hard to reach” populations.

### Limitations

Although we did account for new HIV infections over time within the Markov modeling structure, our cohort model did not include dynamic transmission or account for the potential impact of HIVST on transmission and the underlying community prevalence of HIV over time. An earlier HIV diagnosis has the potential to increase pre-symptomatic treatment and decrease transmissibility (65). Without including dynamic transmission modeling, the cost-utility of HIVST might be underestimated. If HIVST was capable of reducing the underlying community prevalence of HIV, there may be a gradual decrease in new HIV infections and therefore a reduction in ART costs and increased DALYs averted. Alternatively, with a decrease in the test-positivity of HIV (which we found was a driver of cost-effectiveness), the cost-utility of the HIVST intervention could decrease. There is a lack of literature to inform how HIVST might impact long-term community incidence of HIV; thus, it is challenging to predict how changing incidence would impact cost-utility.

We have followed the WHO’s CHOICE economic reporting guidelines (55) and used a WTP threshold based on GDP *per capita*. This does have health equity implications, as an intervention that was cost-effective in South Africa has a much lower chance of being cost-effective in Malawi despite similar efficacy. Recently, various alternatives have been introduced to determine WTP, including using an opportunity cost approach, reporting costs as a percentage of GDP *per capita*, or requiring participants to assign a value to outcome measures using a standard gamble and time trade-off (66, 67). These have been primarily used for QALYs, and DALYs averted continue to be the most common utility outcome used within a global health context. Therefore, to allow generalizability across studies, we have continued to use cost per DALY averted as our primary outcome. The ICERs, costs, and efficacy are presented in addition to conclusions around cost-effectiveness which allows alternate WTP thresholds to be applied.

There are many prevention strategies for HIV transmission (beyond PrEP), that are not specifically included in our model. Since we have reported incremental cost-utility ratios (ICERs), our cost-utility estimates would not be affected by these unless there was a differential uptake of the preventative strategies between the digital-based HIVST cohort and the FBT cohort.

We have used the relevant existing economic data to inform our model, but unfortunately there is a lack of literature around the costs of digital-based HIVST, which has limited the strength of our inputs. Since there were no country-specific studies for either Malawi or South Africa; therefore, we had to generalize from other countries using a ratio of GDP *per capita*. This highlights the need for further cost-effectiveness data from digital-based HIVST initiatives. There was a lack of literature around the effectiveness of DB HIVST that required us to generalize across contexts.

### Key policy implications

As internet access becomes increasingly available across nations, digital-based approaches offer a promising avenue for promoting HIVST while maintaining anonymity. Digital-based HIVST can be offered digitally with websites and applications that focus on key populations, such as MSM dating applications (21). A recent systematic review found that digital-based strategies were associated with a 1.5 times increased rate of HIVST among the MSM population and were perceived as more accessible and convenient (68).

Based on our model, ST (both CB and DB) was cost-effective compared to FBT, but depended on key drivers including underlying HIV test positivity, linkage to care, ART acceptability, and HIVST cost. In areas with low endemic rates of HIV, DB HIVST is most likely to be cost-effective when implemented among key populations with high rates of undiagnosed HIV. Strategies to improve cost-utility include ensuring adequate linkage to confirmatory testing and treatment, negotiating reduced unit test cost, and reducing costs associated with implementation. HIVST implemented with digital-based supports is a key strategy that can improve cost-utility (despite increasing overall costs). It increases accessibility and support linkage to care.

Confidentiality is a major concern with both facility-based and self-testing for HIV. Concerns about recognized in a health care facility while testing for HIV have been reported as perceived benefits of self-testing, which can be done in the privacy of your home (69). Privacy and security in the context of digital-based health care is an increasingly relevant issue as electronic medical records become common. It would need to be considered when implementing a DB HIVST program.

## Conclusion

Self-testing is a promising new strategy that may improve access to diagnosis of infectious disease among hard-to-reach populations. Our model found that the cost-utility of HIVST using DB interventions varied between $769-17839/DALY averted.

As HIV diagnosis and treatment evolve, it becomes increasingly crucial to ensure that marginalized patient populations can also derive benefit from advancements in HIV care. The improved accessibility of digital-based HIVST makes it an appealing strategy for reaching individuals who face barriers to conventional testing. Our model suggests that DB HIVST is cost-effective in a variety of different contexts. Both digital and community-based interventions can increase accessibility to HIVST and support improving the linkage to care and rates of ART initiation (which are key drivers of cost-effectiveness).

## Data Availability

Publicly available datasets were analyzed in this study. This data can be found here: available via prior publications (referenced).

## Supplementary material

The Supplementary material of this article can be found online at: https://www.frontiersin.org/articles/10.3389/fpubh.2024.1440104/full#supplementary-material

Appendix Figure 1 Deterministic Analysis of Digital-Based HIVST in Malawi. This tornado diagram shows that the major drivers of cost-effectiveness of DB HIVST in Malawi were cost of the HIV self-test, underlying HIV test-positivity, linkage to care and the cost of ART. The willingness to pay threshold used for this analysis was 3xGDP per capita ($1230/DALY averted). HIVST, HIV self-testing; DB, digital-based; ART, antiretroviral therapy; WTP, willingness to pay; EV, expected value.

Appendix Figure 2 Deterministic Analysis of Digital-Based HIVST in South Africa. This tornado diagram shows that the major driver of cost-effectiveness of DB HIVST in South Africa was underlying HIV test-positivity. The willingness to pay threshold used for this analysis was 3xGDP per capita ($18003/DALY averted). HIVST, HIV self-testing; DB, digital-based; ART, antiretroviral therapy; WTP, willingness to pay; EV, expected value.

Appendix Figure 3 Deterministic Analysis of Digital-Based HIVST in Brazil. This tornado diagram shows that the major driver of cost-effectiveness of DB HIVST in South Africa was cost of the HIV self-test and linkage to care. The willingness to pay threshold used for this analysis was 3xGDP per capita ($26152/DALY averted). HIVST, HIV self-testing; DB, digital-based; ART, antiretroviral therapy; WTP, willingness to pay; EV, expected value.
