# Supplementary figures and images for: Understanding the cost-utility of implementing HIV self-testing with digital-based supports
Source: Front Public Health. 2025 Jan 14;12:1440104. doi: 10.3389/fpubh.2024.1440104 (PMC11772369; doi:10.3389/fpubh.2024.1440104)

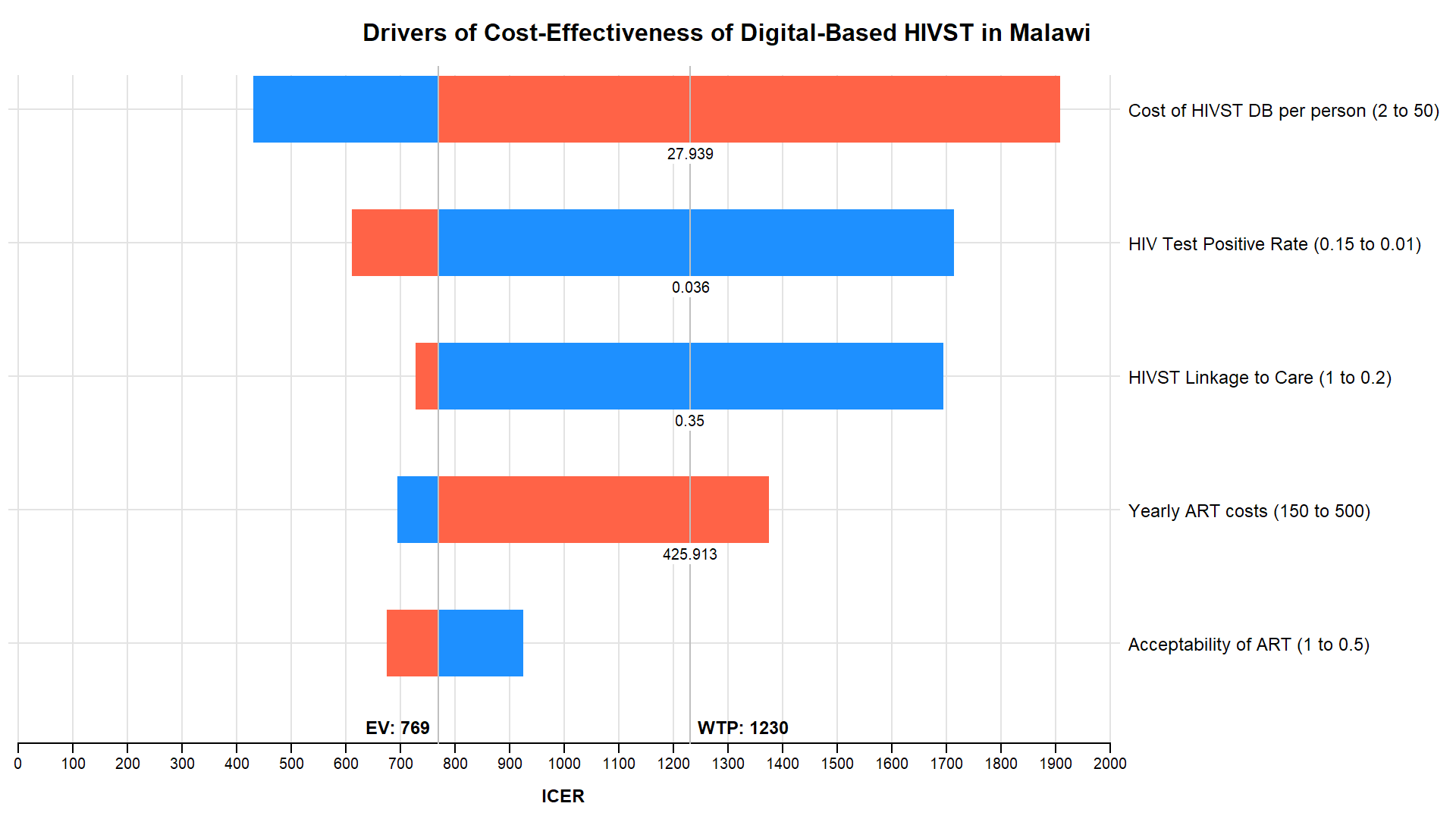

Supplement: Appendix Figure 1 — Deterministic Analysis of Digital-Based HIVST in Malawi. This tornado diagram shows that the major drivers of cost-effectiveness of DB HIVST in Malawi were cost of the HIV self-test, underlying HIV test-positivity, linkage to care and the cost of ART. The willingness to pay threshold used for this analysis was 3xGDP per capita ($1230/DALY averted). HIVST, HIV self-testing; DB, digital-based; ART, antiretroviral therapy; WTP, willingness to pay; EV, expected value. [file Image_1.tif]

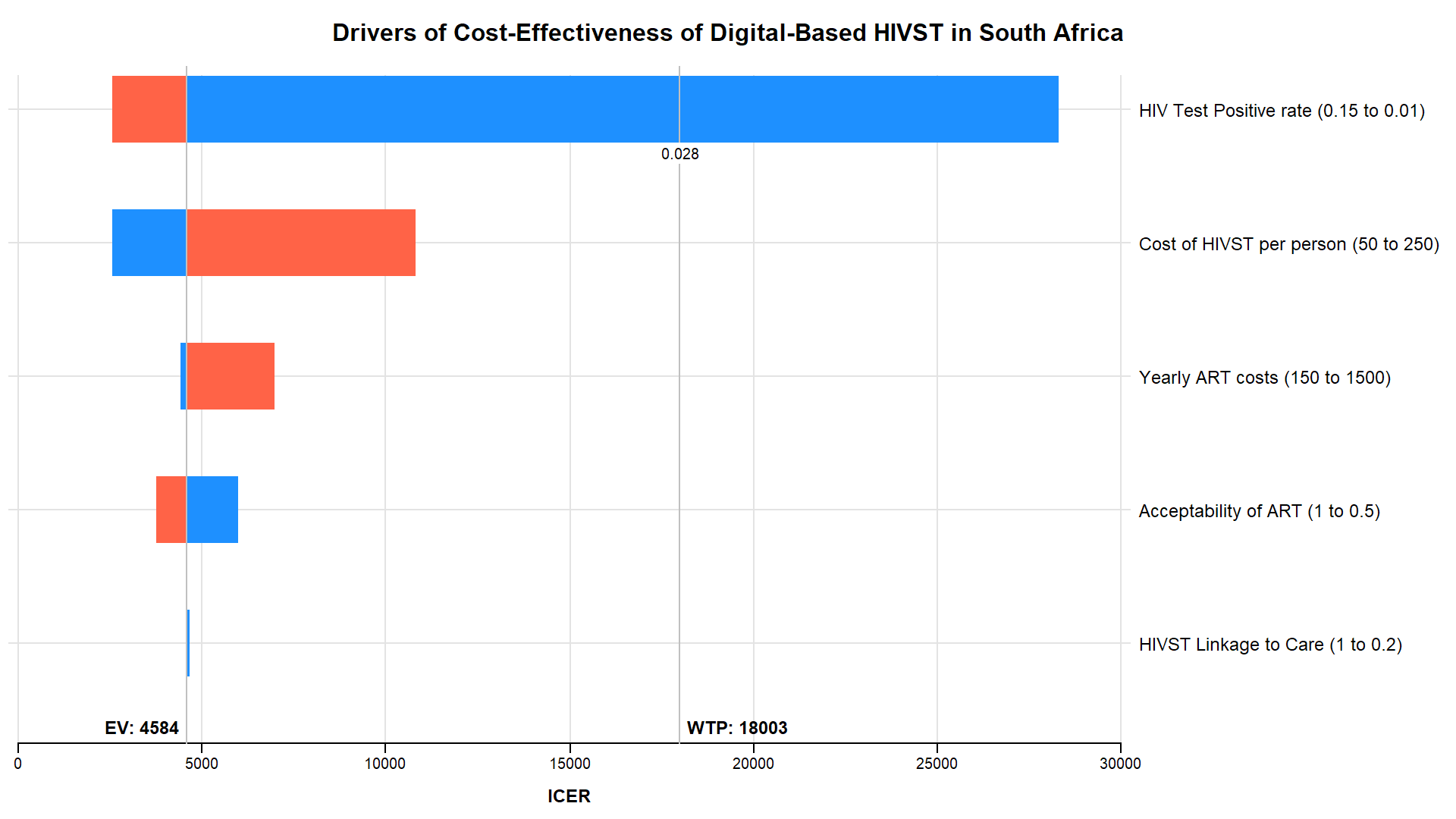

Supplement: Appendix Figure 2 — Deterministic Analysis of Digital-Based HIVST in South Africa. This tornado diagram shows that the major driver of cost-effectiveness of DB HIVST in South Africa was underlying HIV test-positivity. The willingness to pay threshold used for this analysis was 3xGDP per capita ($18003/DALY averted). HIVST, HIV self-testing; DB, digital-based; ART, antiretroviral therapy; WTP, willingness to pay; EV, expected value. [file Image_2.tif]

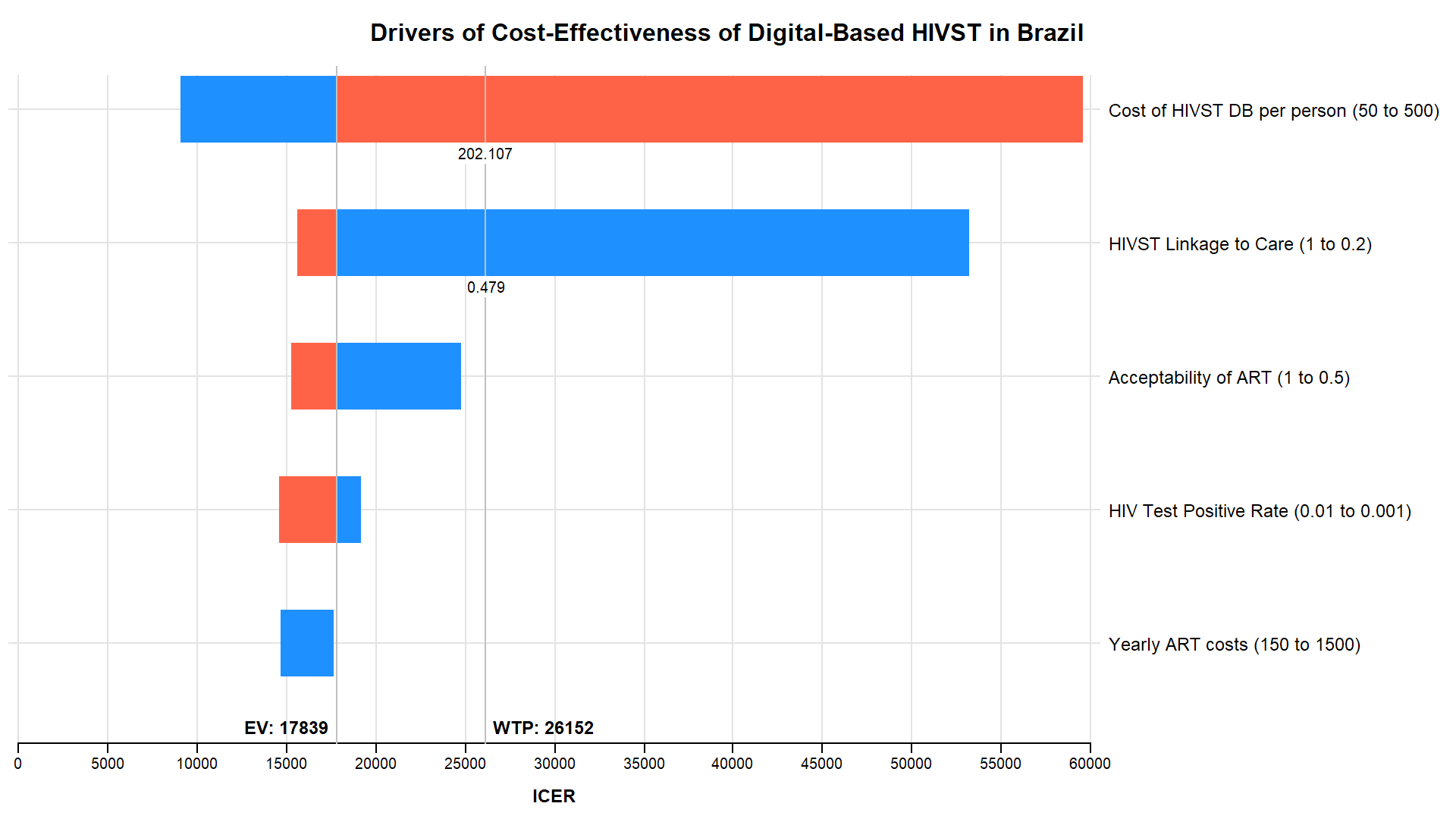

Supplement: Appendix Figure 3 — Deterministic Analysis of Digital-Based HIVST in Brazil. This tornado diagram shows that the major driver of cost-effectiveness of DB HIVST in South Africa was cost of the HIV self-test and linkage to care. The willingness to pay threshold used for this analysis was 3xGDP per capita ($26152/DALY averted). HIVST, HIV self-testing; DB, digital-based; ART, antiretroviral therapy; WTP, willingness to pay; EV, expected value. [file Image_3.tif]
