# Supplementary material for: Understanding the cost-utility of implementing HIV self-testing with digital-based supports
Source: Front Public Health. 2025 Jan 14;12:1440104. doi: 10.3389/fpubh.2024.1440104 (PMC11772369; doi:10.3389/fpubh.2024.1440104)
Supplement: Supplementary file 4 [file Table_1.docx]

Appendices

Model Description:

For our intervention, we have modeled either digital or community-based HIVST in addition to facility-based testing. In this scenario, there would be three possibilities, individuals who accept self-testing, individuals who accept conventional testing and those who do not accept both. Individuals who accept conventional testing would go through a process similar to what is described in the previous paragraph. Individuals who would accept HIV self-testing, will screen either positive or negative. If positive, they would be offered confirmatory testing at a health care facility. Once confirmed positive, they will be counseled on ART therapy and would be started if accepted. Individuals who are lost to follow up before diagnostic testing would go into the HIV positive, untreated Markov state. Those who are confirmed to have HIV but do not accept ART initiation will go into the HIV positive, diagnosed but untreated Markov state.

Our reference strategy is facility base testing scenario, where individuals who choose to be tested would self-present to a facility offering HIV testing and would be offered the conventional rapid HIV test (finger-prick). If positive, they would be offered a confirmatory rapid test (finger-prick). In case of discordant results, an ELISA would be performed. Once confirmed positive, they would be provided with counselling and ART therapy would be initiated if accepted by the patient. Individuals who are lost to follow-up or do not accept ART initiation will go into the HIV positive, diagnosed but untreated Markov state.

The death rate was country specific and included for all Markov states, based on the general population mortality sourced from the World Bank database^1^. The exception to this was the death rate for AIDS, which was higher than that of the general population and based on published studies^2-4^.

Key Model Assumptions:

There are several important assumptions embedded within the model structure. We assumed that all individuals entering the model had an equal probability of testing positive and that the test positivity rate was constant over the model horizon. There was a lack of literature in terms of the impact of HIVST on long term ART adherence. We extrapolated ART acceptability and adherence based on facility-based studies and applied this to the HIVST scenario.

For each cycle the population has a probability of accepting ST or FBT but given the decision tree structure the testing options are mutually exclusive. Therefore, our model cannot account for individuals who may test multiple times per year or test using both ST and FBT approaches. We assumed that previous HIV testing would not impact risk taking behavior or modify the risk of new HIV infection. We assumed universal exposure to the intervention, which may not be realistic particularly for digital-based HIVST (where individuals may not have access to a device or internet). To help account for this we varied both uptake and linkage to care in deterministic analysis.

This model does not include HIV transmission or the epidemiological impact of HIVST on the underlying prevalence of HIV within the community, therefore the underlying HIV test positivity rate remains constant over the thirty- year cycle. If HIVST decreases the prevalence of HIV within a community, then our model will underestimate the cost effectiveness as it will not account for reduced transmission over time.

Description of Sourced Studies

Malawi- Digital-Based HIVST

There were no studies reporting costs conducted within Malawi for digital-based HIVT. Costing and efficacy data were used from a Kenyan study. To adjust for differences in regional costs, a GDP based ratio (Malawian GDP per capita/Kenyan GDP per capita) was applied to costs prior to adjustment for inflation and conversion to USD.

The studies used populate the model with HIV cost and efficacy data were conducted by the same group using the same intervention among two different subpopulations. The interventional studies included were “A Randomized Controlled Trial to Increase HIV Testing Demand Among Female Sex Workers in Kenya Through Announcing the Availability of HIV Self-testing Via Text Message”^5^ and “Offering self-administered oral HIV testing to truck drivers in Kenya to increase testing: a randomized controlled trial”^6^ and costs were sourced from a separately published economic analysis “Costing analysis of an SMS-based intervention to promote HIV self-testing amongst truckers and sex workers in Kenya”^7^.

The intervention used electronic medical record data and sent text messages to truckers and female sex-workers who were registered with health care centers. The text message promoted the standard of care (blood-based HIV testing), enhanced standard of care (with more frequent messaging) or text messages offering both the standard of care and HIV self-testing (at home or in the clinic). This study showed that offering HIVST increased uptake of testing and was cost-effective compared to the standard of care.

Brazil- Digital-Based HIVST

The study used to populate the model with efficacy data was “An Internet-Based HIV Self-Testing Program to Increase HIV Testing Uptake Among Men Who Have Sex With Men in Brazil: Descriptive Cross-Sectional Analysis”^8^, while costs of the intervention were reported by a separate publication “Comprehensive approach to HIV/AIDS testing and linkage to treatment among men who have sex with men in Curitiba, Brazil”^9^.

This study used an Internet-based platform that was linked to a dating website for men who have sex with men. This website promoted HIVST and allowed individuals to register to have tests mailed to their home. Post-test counseling was provided via online modules. Individuals with reactive HIVSTs were directed to a local center for confirmatory testing and further management. This study concluded that HIVST delivered via an online platform was feasible and cost-effective.

South Africa- Digital-Based HIVST

There were no efficacy studies conducted for digital-based HIVT. Costing and efficacy data were used from the Brazilian study (described above). To adjust for differences in regional costs, a GDP based ratio (South African GDP per capita/Brazilian GDP per capita) was applied to costs prior to adjustment for inflation and conversion to USD.

Malawi- Community-Based HIVST

Costing and efficacy data was sourced from Indravudh et al, based on their publication “Pragmatic economic evaluation of community-led delivery of HIV self-testing in Malawi”^10^.

They used community workers to mass-distribute HIVST throughout a community. They achieved 100% distribution within the community. They assessed either standard of care (facility-based HIVST) versus HIVST along with facility-based testing.

They found that HIVST cost was 4.57 USD per person but had a low probability of being cost-effective when cost per new HIV-positive diagnosis was considered. They found that underlying prevalence of HIV positivity was a major driver of cost-effectiveness and concluded that HIVST should be considered in populations with high underlying test-positivity.

Brazil- Community-Based HIVST

The community-based intervention for Brazil is based on a publication by DaCruz et al, “Comprehensive approach to HIV/AIDS testing and linkage to treatment among men who have sex with men in Curitiba, Brazil”^9^. This study assessed the impact of mobile testing units offering HIVST. Reactive tests required follow up at a health care center for confirmatory testing and post-test counseling. They found that mobile testing units were associated with a cost per test of $137 and had a 66% rate of linkage to ongoing care.

South Africa- Community-Based HIVST

The community-based intervention for South Africa is based on a study published by Pettifor et al, “HIV self-testing among young women in rural South Africa: A randomized controlled trial comparing clinic-based HIV testing to the choice of either clinic testing or HIV self-testing with secondary distribution to peers and partners”^11^. This study assessed conventional HIV testing compared to the choice of conventional or HIVST among women aged 18-26. They found that individuals were overwhelmingly more likely to chose HIVST compared to conventional testing, however the benefit of HIVST compared to conventional testing decreased over follow up time.

Facility-Based Testing

Cost and efficacy data for facility-based testing arm of our model was sourced from an extensive literature review that evaluated all existing economic literature for HIVST. An unweighted mean costs including standard deviations was calculated from 22 included studies for facility-based testing.

Facility-based testing required an individual to be tested at a health care center by another person, followed by country-specific guidelines for confirmatory testing and treatment.

1. DataBank WB. Data from: Death Rate, crude per 1,000 people 2020.

2. Fox GJ, Barry SE, Britton WJ, Marks GB. Contact investigation for tuberculosis: a systematic review and meta-analysis. *Eur Respir J*. Jan 2013;41(1):140-56. doi:10.1183/09031936.00070812

3. Mindel A, Tenant-Flowers M. ABC of AIDS: Natural history and management of early HIV infection. *BMJ*. May 26 2001;322(7297):1290-3. doi:10.1136/bmj.322.7297.1290

4. Vellozzi C, Brooks JT, Bush TJ, et al. The study to understand the natural history of HIV and AIDS in the era of effective therapy (SUN Study). *Am J Epidemiol*. Mar 1 2009;169(5):642-52. doi:10.1093/aje/kwn361

5. Kelvin EA, George G, Mwai E, et al. A Randomized Controlled Trial to Increase HIV Testing Demand Among Female Sex Workers in Kenya Through Announcing the Availability of HIV Self-testing Via Text Message. *AIDS Behav*. Jan 2019;23(1):116-125. doi:10.1007/s10461-018-2248-5

6. Kelvin EA, George G, Mwai E, et al. Offering self-administered oral HIV testing to truck drivers in Kenya to increase testing: a randomized controlled trial. *AIDS Care*. Jan 2018;30(1):47-55. doi:10.1080/09540121.2017.1360997

7. George G, Chetty T, Strauss M, et al. Costing analysis of an SMS-based intervention to promote HIV self-testing amongst truckers and sex workers in Kenya. *PLoS One*. 2018;13(7):e0197305. doi:10.1371/journal.pone.0197305

8. De Boni RB, Veloso VG, Fernandes NM, et al. An Internet-Based HIV Self-Testing Program to Increase HIV Testing Uptake Among Men Who Have Sex With Men in Brazil: Descriptive Cross-Sectional Analysis. *J Med Internet Res*. Aug 1 2019;21(8):e14145. doi:10.2196/14145

9. da Cruz MM, Cota VL, Lentini N, et al. Comprehensive approach to HIV/AIDS testing and linkage to treatment among men who have sex with men in Curitiba, Brazil. *PLoS One*. 2021;16(5):e0249877. doi:10.1371/journal.pone.0249877

10. Indravudh PP, Fielding K, Kumwenda MK, et al. Effect of community-led delivery of HIV self-testing on HIV testing and antiretroviral therapy initiation in Malawi: A cluster-randomised trial. *PLoS Med*. May 2021;18(5):e1003608. doi:10.1371/journal.pmed.1003608

11. Pettifor A, Lippman SA, Kimaru L, et al. HIV self-testing among young women in rural South Africa: A randomized controlled trial comparing clinic-based HIV testing to the choice of either clinic testing or HIV self-testing with secondary distribution to peers and partners. *EClinicalMedicine*. Apr 2020;21:100327. doi:10.1016/j.eclinm.2020.100327
